# Visualization of breast cancer-related protein synthesis from the perspective of bibliometric analysis

**DOI:** 10.1186/s40001-023-01364-4

**Published:** 2023-10-27

**Authors:** Jiawei Xu, Chengdong Yu, Xiaoqiang Zeng, Weifeng Tang, Siyi Xu, Lei Tang, Yanxiao Huang, Zhengkui Sun, Tenghua Yu

**Affiliations:** 1 Department of Breast Surgery, Affiliated Cancer Hospital of Nanchang University, Jiangxi Cancer Hospital, The Second Affiliated Hospital of Nanchang Medical College, Jiangxi Clinical Research Center for Cancer, Nanchang, Jiangxi Province, 330029 China; 2 Fuzhou Medical College of Nanchang University, Fuzhou, 344000 China

**Keywords:** Breast cancer, Bibliometric analysis, Protein synthesis

## Abstract

Breast cancer, as a daunting global health threat, has driven an exponential growth in related research activity in recent decades. An area of research of paramount importance is protein synthesis, and the analysis of specific proteins inextricably linked to breast cancer. In this article, we undertake a bibliometric analysis of the literature on breast cancer and protein synthesis, aiming to provide crucial insights into this esoteric realm of investigation. Our approach was to scour the Web of Science database, between 2003 and 2022, for articles containing the keywords “breast cancer” and “protein synthesis” in their title, abstract, or keywords. We deployed bibliometric analysis software, exploring a range of measures such as publication output, citation counts, co-citation analysis, and keyword analysis. Our search yielded 2998 articles that met our inclusion criteria. The number of publications in this area has steadily increased, with a significant rise observed after 2003. Most of the articles were published in oncology or biology-related journals, with the most publications in Journal of Biological Chemistry, Cancer Research, Proceedings of the National Academy of Sciences of the United States of America, and Oncogene. Keyword analysis revealed that “breast cancer,” “expression,” “cancer,” “protein,” and “translation” were the most commonly researched topics. In conclusion, our bibliometric analysis of breast cancer and related protein synthesis literature underscores the burgeoning interest in this research. The focus of the research is primarily on the relationship between protein expression in breast cancer and the development and treatment of tumors. These studies have been instrumental in the diagnosis and treatment of breast cancer. Sustained research in this area will yield essential insights into the biology of breast cancer and the genesis of cutting-edge therapies.

## Introduction

Breast cancer is the most common cancer in women worldwide and the number of patients increased year by year.[1]. It is a complex disease that can be caused by a variety of factors, including genetic mutations, hormonal imbalances, and lifestyle choices, even Job authority [2–8]. The most common risk factors for breast cancer include age, gender, inherited risk of breast cancer, reproductive history, and exposure to certain chemicals and radiation [9]. One of the key factors in the development and progression of breast cancer is the overexpression or abnormal synthesis of certain proteins in breast tissue [10–12]. These proteins include estrogen receptor (ER), progesterone receptor (PR), and human epidermal growth factor receptor 2 (HER2) [13–15]. ER and PR are hormones that play important roles in breast tissue development and function [16]. When breast cancer cells overexpress these hormones or their receptors, they can become more aggressive and resistant to treatment [17–20]. HER2 is a protein that helps regulate cell growth and division. When HER2 is overexpressed in breast cancer cells, it can lead to uncontrolled cell growth and tumor formation [15, 21–23].

Protein synthesis has emerged as a pivotal research area in the battle against breast cancer [24]. Aberrant protein synthesis represents a hallmark feature of breast cancer, and a comprehensive understanding of the underlying mechanisms is critical for the development of targeted therapeutic interventions capable of effectively managing this disease [25–27]. In the past twenty years, significant progress has been made in identifying key proteins involved in breast cancer [28–32]. Through in-depth studies of protein synthesis related to breast cancer, researchers have been able to improve the effectiveness of treatment for patients with breast cancer [33–40]. However, much work remains to be done in this area, as many breast cancer patients still do not respond to current treatments. Advances in protein synthesis research hold great promise for developing more effective therapies and improving outcomes for all breast cancer patients [41–44]. By understanding the complex interplay between protein synthesis and breast cancer, researchers can continue to make significant progress in treatment of breast cancer patients.

Breast cancer research related to protein synthesis is a complex topic, and its intricate molecular mechanisms are not easily comprehensible. However, the potential clinical applications of this research are broad, and thus, further investigation is warranted to unravel the perplexing interplay between protein synthesis and breast cancer. A consensus is growing among researchers that analyzing protein synthesis is pivotal to comprehending the development and progression of breast cancer [45–47]. The design of effective therapeutic strategies also hinges on a deeper understanding of this perplexing topic.

In the realm of breast cancer-related protein synthesis, a distinct order of priority governs the current research focus. Nevertheless, the field remains contentious, with unresolved debates concerning relevant conclusions [48, 49]. Surprisingly, a dearth of studies explores the existing literature from the vantage point of bibliometric analysis. Furthermore, the majority of scholars' investigations in this domain tend to limit themselves to comprehensive literature reviews and personal experience summaries, thereby neglecting the essential elements of completeness and macroscopicity.

The study of breast cancer-related protein synthesis has also seen a growth in research publications over the years. The available literature on this topic has likely increased with advancements in technology, allowing researchers to generate and disseminate information more rapidly. Consequently, keeping up with the vast amount of literature through manual reading has become challenging, necessitating the use of quantitative approaches to complement qualitative analyses [50, 51].

The accelerated pace of scientific knowledge doubling, combined with the availability of research literature in digital formats, has contributed to the advancement of research in the field of breast cancer-related protein synthesis. The integration of qualitative and quantitative methods can aid in synthesizing and understanding the evolving body of scientific evidence and knowledge in this crucial area of breast cancer research.

Bibliometrics is a field of inquiry that employs various quantitative methods to assess the impact of scientific publications [52–55]. The primary objective of bibliometric studies is to measure the influence of scientific research while simultaneously identifying the intricate patterns and trends inherent in scientific production [56–58]. It is noteworthy that the extensive arsenal of bibliometric analysis offers a range of applications in answering research questions, including but not limited to, the identification of authors or articles that have garnered substantial attention, the cartography of research topics and their evolution over time, the measurement of the consequential impact and widespread visibility of scientific journals, and the analysis of complex collaborations among researchers and institutions [59–64].

In the realm of medical research, bibliometrics has experienced a remarkable surge in popularity [65, 66]. Its implementation in the field of medicine has yielded fruitful outcomes, as evidenced by studies indicating a positive literature production trend. Notably, this trend stands on par with the general bibliometric trend and even surpasses the broader trend observed in medicine [67]. Bibliometrics has proven its significance in various research areas, including molecular biology, immunology, genetics, and cancer [68–73]. Notably, bibliometric analysis has made substantial contributions to disease research [74–76]. However, a notable gap exists in the assessment of scientific results in the field of breast cancer-associated protein synthesis. Therefore, it is crucial to investigate the current status of research on breast cancer-associated protein synthesis.

The use of bibliometric analysis in breast cancer research is an innovative and powerful tool that enables researchers to identify key areas of investigation, prominent researchers, and emerging trends [77–79]. As the volume of scientific literature continues to expand at a breathtaking pace, bibliometric analysis provides a structured and objective approach to navigate the ever-growing body of knowledge in this field [80–86].

CiteSpace and VOSviewer are two popular software programs that are commonly used for bibliometric analysis [87–91]. By leveraging both CiteSpace and VOSviewer, researchers can establish an intricate network of co-citation links among articles focused on breast cancer-related protein synthesis. The output of this meticulous process yields a thorough visualization and analysis of the scientific literature, thereby easing the identification of crucial research areas and significant authors [92–94]. The added value to the procedure is apparent through the visualization of co-authorship networks and identification of research clusters [95–97]. The utilization of this approach empowers researchers to achieve a superior comprehension of the literature surrounding breast cancer-related protein synthesis and identify prospects for further research.

In conclusion, the study of protein synthesis in breast cancer is a rapidly evolving area of research that holds immense promise for improving the diagnosis, treatment, and ultimately, the survival rates of patients with breast cancer [98–101]. The use of bibliometric analysis is an indispensable tool in this endeavor, enabling researchers to make sense of the vast and complex literature on this topic. We hope that our analysis will inspire further research in this exciting field and ultimately contribute to the eradication of breast cancer.

### Aim of the study

This research aims to identify the importance of breast cancer-related protein synthesis and address the following research questions.

1. What is the publication output trend in breast cancer-related protein synthesis research?
2. Which journals have been the most influential in publishing articles on this topic?
3. What are the top institutions and countries contributing to breast cancer-related protein synthesis research?
4. Who are the most prominent authors in this field and what are their contributions?
5. What are the key areas of focus in breast cancer-related protein synthesis research based on keyword analysis?
6. What are the potential hotspots and frontiers in breast cancer-related protein synthesis research, based on keyword bursts?

Previously, a comprehensive and structured analysis of the scientific literature on protein synthesis in breast cancer was lacking. However, this study successfully addresses this gap by utilizing bibliometric techniques to systematically analyze and present the research landscape in this field. This study offers crucial insights into breast cancer-related protein synthesis research activity, including the number of publications over time, influential journals, top institutions and countries involved, highly cited authors, co-citation relationships, and prominent keywords with their burst strength. This study sheds light on current focus areas and areas of interest in breast cancer-related protein synthesis research, which can further contribute to the understanding, diagnosis, and treatment of breast cancer.

## Material and methods

### Data source

We utilized the Web of Science Core Collection (WOSCC) as our database for this study. The search was conducted on February 18, 2023.

### Time range

We conducted our search from January 1, 2003 to December 31, 2022.

### Inclusion criteria

We considered all research articles published in the English language that investigated the relationship between breast cancer-related protein synthesis for inclusion in our study.

### Exclusion criteria

For exclusion criteria, this study excluded articles on breast cancer-related protein synthesis that were not published between 2003 and 2022. Furthermore, articles published in a non-English format and non-research articles between 2003 and 2022 were also excluded.

#### Search strategy

**Fig. 1:**
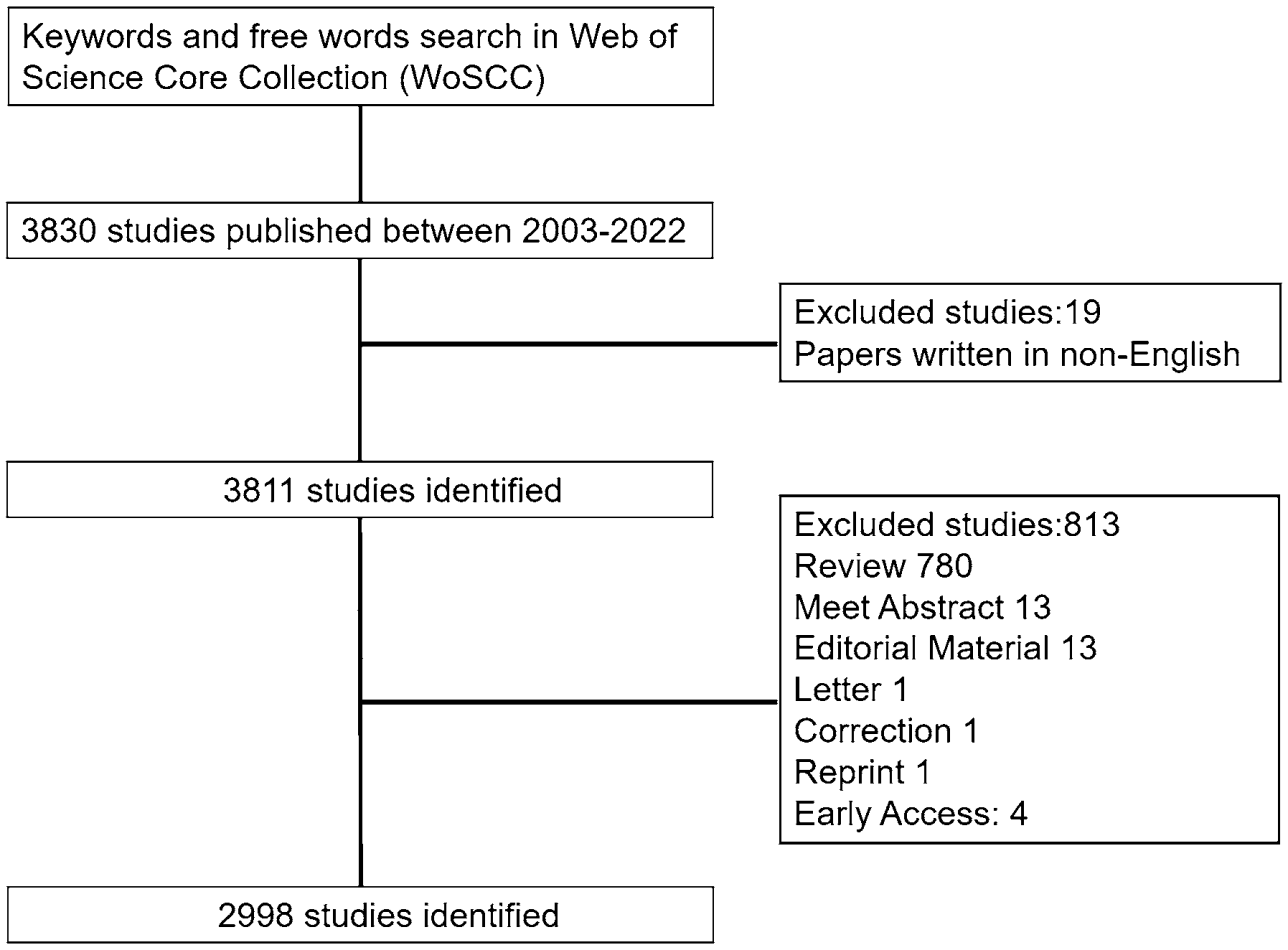
Flow diagram of screening process related to breast cancer-related protein synthesis from the perspective of bibliometric analysis

TS = (((TS = (Breast Neoplasm) OR TS = (Neoplasm, Breast) OR TS = (Breast Tumors) OR TS = (Breast Tumor) OR TS = (Tumor, Breast) OR TS = (Tumors, Breast) OR TS = (Neoplasms, Breast) OR TS = (Breast Cancer) OR TS = (Cancer, Breast) OR TS = (Mammary Cancer) OR TS = (Cancer, Mammary) OR TS = (Mammary Cancers) OR TS = (Malignant Neoplasm of Breast) OR TS = (Breast Malignant Neoplasm) OR TS = (Breast Malignant Neoplasms) OR TS = (Malignant Tumor of Breast) OR TS = (Breast Malignant Tumor) OR TS = (Breast Malignant Tumors) OR TS = (Cancer of the Breast) OR TS = (Mammary Carcinoma, Human) OR TS = (Cancer of Breast) OR TS = (Carcinoma, Human Mammary) OR TS = (Carcinomas, Human Mammary) OR TS = (Human Mammary Carcinomas) OR TS = (Mammary Carcinomas, Human) OR TS = (Human Mammary Carcinoma) OR TS = (Mammary Neoplasms, Human) OR TS = (Human Mammary Neoplasm) OR TS = (Human Mammary Neoplasms) OR TS = (Neoplasm, Human Mammary) OR TS = (Neoplasms, Human Mammary) OR TS = (Mammary Neoplasm, Human) OR TS = (Breast Carcinoma) OR TS = (Breast Carcinomas) OR TS = (Carcinoma, Breast) OR TS = (Carcinomas, Breast)) AND ((TS = (Protein Biosynthesis) OR TS = (Biosynthesis, Protein) OR TS = (Translation, Genetic) OR TS = (mRNA Translation) OR TS = (Translation, mRNA) OR TS = (mRNA Translations) OR TS = (Protein Translation) OR TS = (Translation, Protein) OR TS = (Ribosomal Peptide Biosynthesis) OR TS = (Protein Biosynthesis, Ribosomal) OR TS = (Biosynthesis, Ribosomal Protein) OR TS = (Ribosomal Protein Biosynthesis) OR TS = (Protein Synthesis, Ribosomal) OR TS = (Ribosomal Protein Synthesis) OR TS = (Synthesis, Ribosomal Protein) OR TS = (Genetic Translation) OR TS = (Genetic Translations) OR TS = (Peptide Biosynthesis, Ribosomal) OR TS = (Biosynthesis, Ribosomal Peptide))) The detailed data retrieval strategies and inclusion procedure of this study are shown in Fig. 1.

#### Data standardization

Before conducting the analysis, we applied the VOSviewer software and made use of its “VOSviewer thesaurus file” feature to standardize the data. This process was essential to ensure consistency in fields such as Authors and Organizations, as the raw data obtained from WoS may exhibit variations and lack uniformity.

#### Ethical consent

Ethical approval was not required as this study does not involve animals or experiments.

### Data analysis and visualization

VOSviewer and CiteSpace are recognized as the primary software tools used for bibliometric analysis. In 2009, Eck and Waltman from Leiden University developed VOSviewer, a program specifically designed for constructing scientometrics networks and visualizing knowledge maps [102]. VOSviewer, well known for its powerful visual capabilities, provides a direct representation of collaborative associations among research topics [103–105]. The size of each node corresponds to the frequency of co-occurrence, and the lines connecting nodes indicate their co-occurrence relationships, with colors indicating clustering patterns. CiteSpace, a Java application specifically designed for bibliometric analysis, was developed by Chen Chaomei [106, 107]. This software excels in aggregating keyword bursts, effectively highlighting emerging trends and dynamic shifts within research hotspots [108, 109].

In this investigation, we utilized the VOSviewer 1.6.19 software to perform a comprehensive visual analysis of authors, institutions, and countries by importing the data as plain text files. Notably, VOSviewer stands out as a widely utilized software tool thoughtfully tailored for bibliometric analysis and visualization. The generated network visualization maps elegantly furnish in-depth insights into the publications’ origins, spanning diverse countries and institutions, while also shedding light on their collaborative relationships. In the generated graph, countries or institutions are represented by nodes, with their sizes corresponding to the number of publications they have contributed. Larger nodes indicate a higher number of publications produced. The graph’s links illustrate the level of association or collaboration between countries or institutions. Remarkably, thicker links signify a stronger degree of collaboration between the respective entities.

The color coding of nodes in the VOSviewer network map serves a pivotal purpose, as it encapsulates the clustering of nodes based on their interconnections and relationships within the network. Nodes sharing common ties are allocated identical colors, signifying their affiliation to the same cluster. Clusters, in this context, refer to sets of nodes that manifest a higher density of connections among each other compared to those outside the cluster.

Leveraging clustering techniques, VOSviewer facilitates the discernment of distinct research communities or thematic groups inherent in the network. Each cluster represents a cohesive assemblage of closely related publications or authors, which can indicate collaborative research endeavors, shared topical interests, or comparable methodologies in their scholarly work. Deeper comprehension of these clusters affords valuable insights into the underlying patterns and dynamics of scientific collaborations and knowledge dissemination within the field.

In the context of our specific analysis, the color-coded nodes featured in the network map correspond to various research communities or thematic groups that have emerged from our examination of collaboration and co-citation networks. Through a comprehensive analysis of these clusters, we have gained a profound understanding of the overarching structure of the scholarly landscape and identified pivotal areas of research interest and collaboration.

The VOSviewer software was utilized to generate a keyword network map of breast cancer-related protein synthesis. Subsequently, the obtained map was further analyzed and segmented using Pajek software. The segmentation process involved dividing topics with similar clusters into the same area, facilitating a more organized and comprehensive understanding of the data. Pajek is a sophisticated software application designed for the analysis and visualization of large-scale networks [110]. It is widely employed in network science and allied disciplines to investigate the intricate structures and properties of complex networks. The network map, generated through the utilization of VOSviewer software, is stored in three file formats: .net, .clu, and .vec. Firstly, import the aforementioned file formats into Pajek software and employ the “Draw” function. Subsequently, select “Network + First Partition + First Vector” and activate the “Layers” option. Opt for the “In *y* Direction + Random in *x*” configuration and proceed to execute the “Export” operation. Upon successful export, access the “2D” view and utilize the “send to VOSViewer” function to transfer the exported atlas to VOSviewer. Within VOSviewer, access the “Analysis” feature and apply the “Rotate” function to align the plots accurately, thus obtaining the final graphical representation.

To conduct the visual analysis of keywords and the dual-map overlay of journals, we utilized the advanced software CiteSpace 6.1.R4 (64-bit). The software's parameters were configured as follows: Time slicing was set from January 1, 2003 to December 31, 2022 with a time slice interval of *n* = 1. Additionally, we applied pruning methods, including pathfinder, pruning sliced networks, and pruning the merged networks. Other parameter settings were kept at their default configurations as specified by the software. In our study, we employed the dual-map analysis feature in CiteSpace 6.1R4 to seamlessly integrate the citation data of the primary literature collection onto the foundational graph layer. Subsequently, we conducted a meticulous normalization using Z-Score. Delving deeper, we diligently scrutinized the citation interconnections between the core literature collection and the referenced literature set, enabling a comprehensive analysis of the knowledge absorption patterns pertaining to breast cancer-related protein synthesis.

We harnessed the power of the dual-map overlay technique to illustrate the citation dynamics between two distinct sets of journals. The left side of the visualization showcases the citing journals, while the right side portrays the cited journals. The colored lines artfully connect these nodes, effectively depicting the citation paths between them. Within this dual-map overlay, we introduced two vital parameters: *z* and *f*. The “*z*” value assumes a pivotal role by denoting the node’s cluster fraction along the citation path. Essentially, it quantifies the density of the cluster to which the respective node belongs. On the other hand, the “*f*” value corresponds to the literature count attributed to the node. It represents the number of literature instances associated with a particular node in the overlay path. By considering these critical parameters, we were able to discern the strength of relationships and the extent of impact certain nodes possessed within the broader context of the research landscape. This comprehensive analysis facilitated a deeper understanding of the significance of breast cancer-related protein synthesis in the scholarly domain. Our exploration aims to unravel the intricate knowledge absorption patterns and interrelations among the journals pertinent to our research on breast cancer-related protein synthesis by thoughtfully incorporating these essential factors.

### Screening process

The literature screening process comprised multiple stages. Initially, the titles and abstracts of the retrieved studies were meticulously screened to eliminate any irrelevant contributions. Subsequently, we diligently examined the articles of the selected studies to evaluate their pertinence to our research question. The screening and searching procedures were undertaken independently by three authors (Jiawei Xu, Weifeng Tang, Xiaoqiang Zeng). In instances of dissent, resolution was diligently sought through rigorous discussions or by soliciting the input of the fourth author (Siyi Xu).

## Results

### Publications

**Fig. 2:**
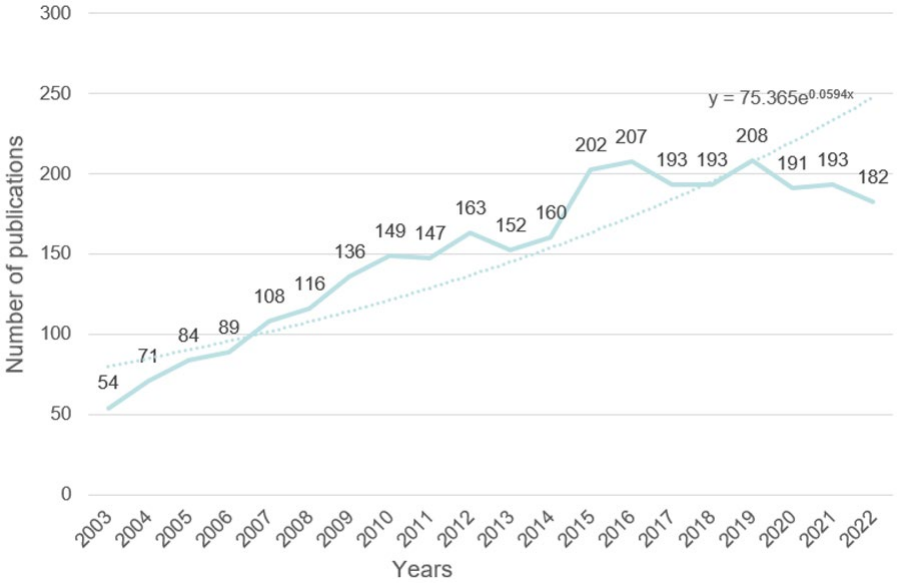
The dynamics of publications related to breast cancer-related protein synthesis for the period 2003 to 2022

Figure 2 presents an illuminating visual representation of the number of published papers focused on the intersection of breast cancer and protein synthesis, spanning the years 2003 to 2022. As a time series analysis of the data reveals, there has been a general upward trend in the number of publications over time, with some notable fluctuations in specific years. It is striking to note that the year 2019 saw a dramatic spike in the number of publications, with a staggering 208 papers dedicated to this crucial area of research. In stark contrast, the year 2003 witnessed a paltry 54 publications, highlighting the vast discrepancy in the attention paid to this field in the early years compared to the current climate of robust research.

Upon further examination, it becomes evident that the trend of the number of published papers over the years manifests as a sawtooth pattern, with an overall increase from 2003 to 2015, punctuated by some noteworthy fluctuations. The data then plateau for a brief period before experiencing a slight dip in recent years, signifying the ebb and flow of the research agenda in this field. Nevertheless, this analysis suggests that the scientific community has consistently maintained an active and unwavering interest in the study of breast cancer and protein synthesis over the past two decades, culminating in a noteworthy surge in publications in the mid-2010s.

### Journal analysis

The WoSCC search showed that the 2998 papers included in the current analysis were published in 777 different journals over the last 20 years. VOSviewer was used to analyze the influence of journals. The top 10 most cited journals are listed in Table 1. Among them, eight publishers are located in the USA, while the other two are located in the United Kingdom. The Journal of Biological Chemistry, which demonstrated the highest number of total citations (7014) with an impact factor of 5.485, ranked first in the research field of breast cancer-related protein synthesis.

**Table 1:** The top 10 most cited journals of breast cancer-related protein synthesis for the period 2003 to 2022

Rank	Journal	Cite	JCR	IF (2021)
1	J Biol Chem	7014	Q2	5.485
2	Cancer Res	6526	Q1	13.312
3	P Natl Acad Sci USA	4587	Q1	12.779
4	Oncogene	3933	Q1	8.756
5	Nature	3661	Q1	69.504
6	Cell	3444	Q1	66.580
7	Nucleic Acids Res	2435	Q1	19.160
8	Science	2369	Q1	63.832
9	Mol Cell Biol	2353	Q2	5.094
10	Clin Cancer Res	2263	Q1	13.801

### Dual-map analysis

**Fig. 3:**
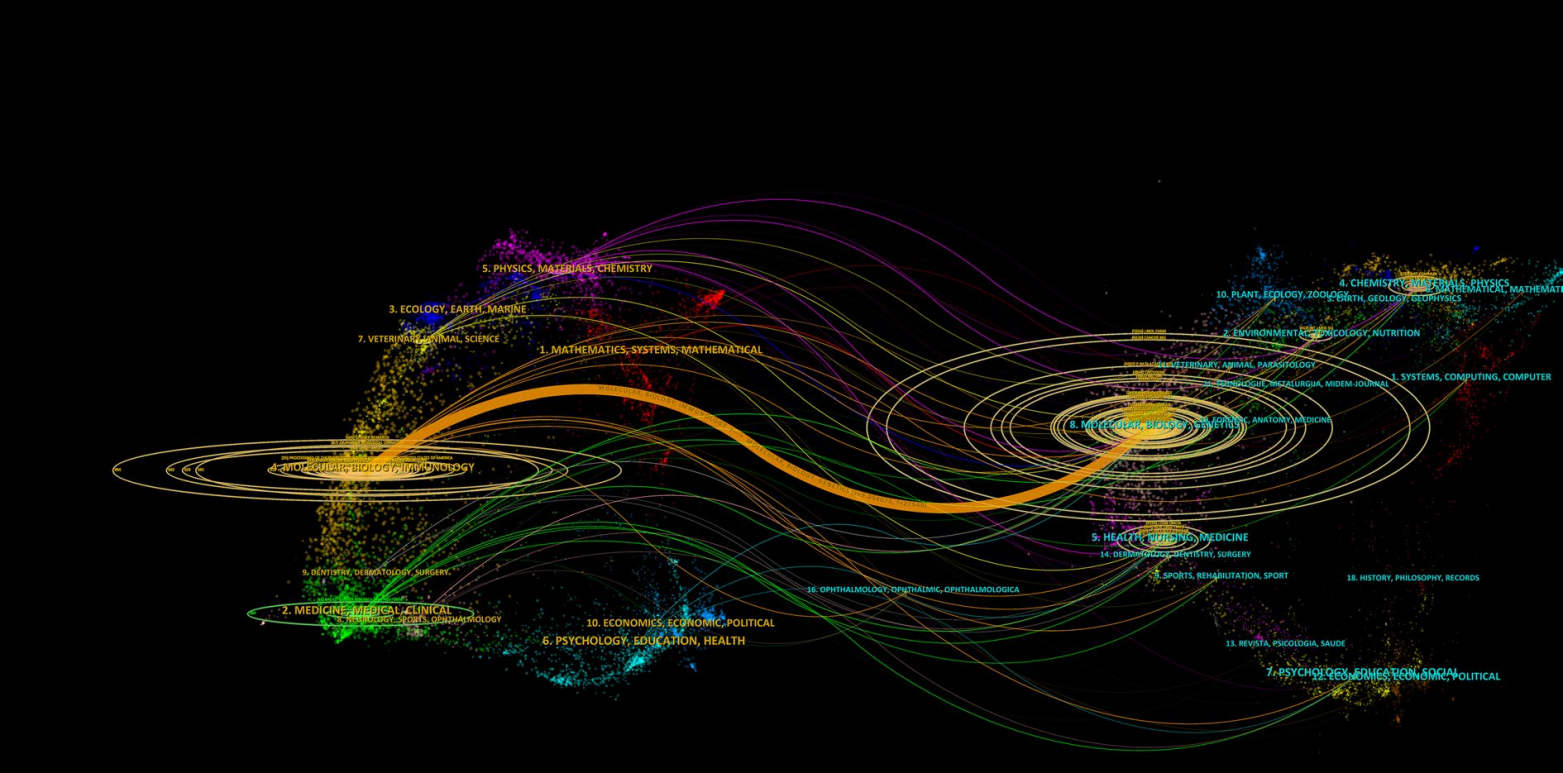
The dual-map overlay of journals on breast cancer-related protein synthesis

The dual-map overlay of journals shows the citation relationship between molecular biology immunology journals (citing journals, presented on the left) and molecular biology genetics journals (cited journals, presented on the right), with the colored line connecting them indicating the citation paths (Fig. 3). The orange-colored line in the figure suggests that articles published in molecular biology genetics journals were primarily cited by articles in molecular biology immunology journals, indicating a strong citation relationship between these two fields.

### Institutions, countries analysis

VOSviewer is a popular software used for bibliometric analysis and visualization. The visualization network maps generated by VOSviewer display information about the number of publications from different countries and institutions, as well as the collaboration between them. The nodes in the graph represent the countries or institutions, with the size of the nodes reflecting the number of publications they have produced. The larger the node, the more publications that country or institution has produced. The links in the graph show the association or collaboration between the countries or institutions. Thicker links indicate stronger collaboration between them.

**Fig. 4:**
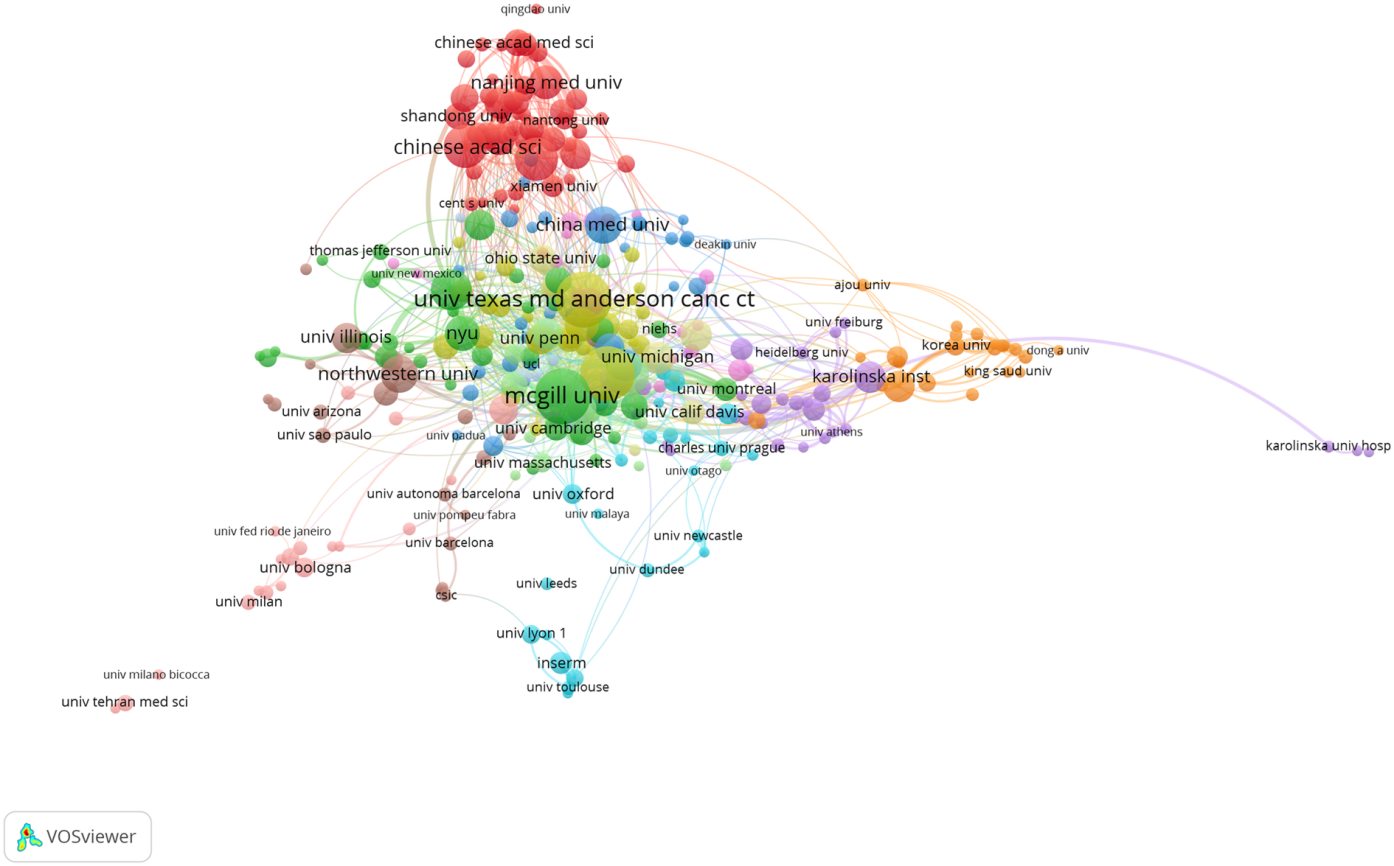
Collaboration network map of institutions of breast cancer-related protein synthesis for the period 2003 to 2022

Fig. 4 shows that certain institutions have emerged as leaders in this field. McGill University, The University of Texas MD Anderson Cancer Center, and the National Cancer Institute have all demonstrated remarkable achievements in breast cancer-related protein synthesis research. This is evident from the larger node sizes and thicker lines in the data, which suggest higher publication numbers and collaboration levels.

The study of breast cancer-related protein synthesis has attracted the attention of numerous academic institutions, including universities, research institutes, and medical centers from across the globe. These institutions bring with them a wealth of knowledge and expertise, which is reflected in the top 10 institutions listed in Table 2.

**Table 2:** The top 10 institutions of breast cancer-related protein synthesis for the period 2003 to 2022

Rank	Institution	Publications	Citations	Average citations	Country
1	McGill Univ	60	5182	86.37	Canada
2	Univ Texas MD Anderson Canc Ctr	60	2902	48.37	USA
3	NCI	57	2666	46.77	USA
4	Harvard Univ	41	4583	111.78	USA
5	Chinese Acad Sci	40	1280	32	China
6	Sun Yat Sen Univ	40	1823	45.58	China
7	Univ Toronto	38	3343	87.97	Canada
8	China Med Univ	33	1036	31.39	China
9	Northwestern Univ	33	1578	47.82	USA
10	NYU	30	1977	65.9	USA

Among them, five institutions are located in the USA, while the other five institutions are located in China and Canada, respectively. At the forefront of this list is McGill University, a leading academic institution located in Canada. With a total of 60 publications and an impressive citation count of 5182, McGill University has established itself as a driving force in this field of research. Harvard University’s research on breast cancer-related protein synthesis has resulted in 41 publications with an outstanding citation count of 4583, making Harvard the global leader in this field. Its average citation count per publication, 111.78, is the highest in the world.

**Fig. 5:**
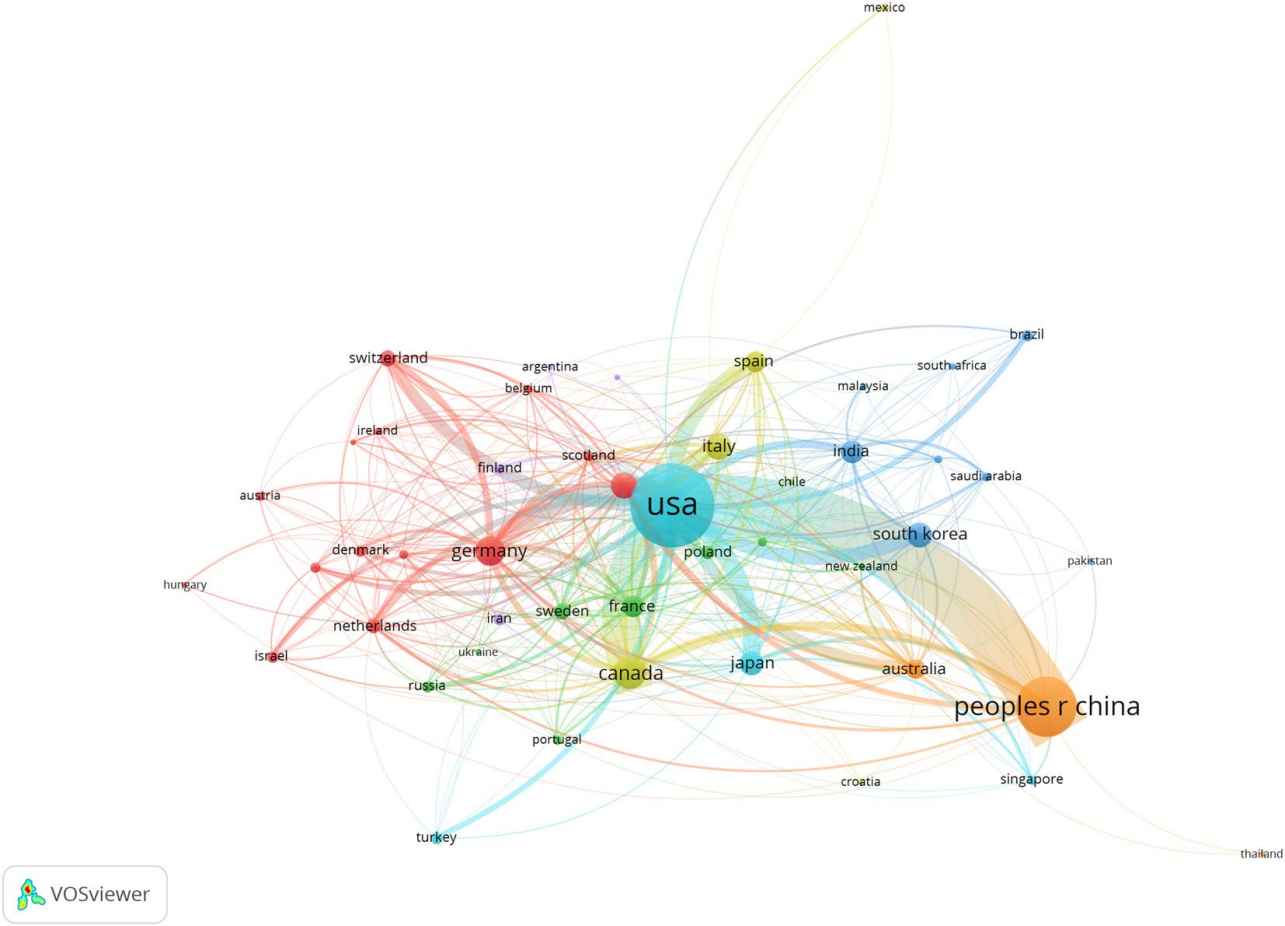
Collaboration network map of countries of breast cancer-related protein synthesis for the period 2003 to 2022

Fig. 5 indicates that the USA, China, and Canada are the leading countries in breast cancer-related protein synthesis research, as they have larger node sizes and thicker lines, which suggest higher publication numbers and collaboration levels. The top countries spearheading the investigation of breast cancer-related protein synthesis evince an unmistakable manifestation of a global research endeavor (Table 3). The leading nations spearheading this investigation have left an indelible mark on the global research landscape. Among these countries, the United States has cemented its position at the forefront of this field, boasting an impressive corpus of 1,218 publications. Not to be outdone, China has emerged as a major player in this space, with a commendable tally of 721 publications that positions it as a strong contender for the top spot. However, the global effort to unravel the mysteries of breast cancer-related protein synthesis extends beyond just these two countries. Canada, Germany, and England trail behind, occupying the third, fourth, and fifth slots with a creditable 216, 179, and 148 publications, respectively. The presence of countries such as South Korea, Japan, and India within the top ten is a clear indication of the worldwide interest in and commitment to this critical area of research. Moreover, the diverse composition of countries in the top ten underscores the importance of international collaboration in expediting research and development. By sharing knowledge and insights, researchers from different countries and cultures can uncover breakthroughs that lead to better diagnosis and treatment of breast cancer.

**Table 3:** The top 10 countries of breast cancer-related protein synthesis for the period 2003 to 2022

Rank	Country	Publication	Cite	Average citation
1	USA	1218	57,650	47.33
2	Peoples R China	669	17,983	26.88
3	Canada	216	11,434	52.94
4	Germany	179	5910	33.02
5	England	148	5299	35.80
6	Italy	144	3735	25.94
7	South Korea	129	3274	25.38
8	Japan	126	5367	42.60
9	India	108	2230	20.65
10	France	104	3385	32.55

### Authors and co-cited authors analysis

**Fig. 6:**
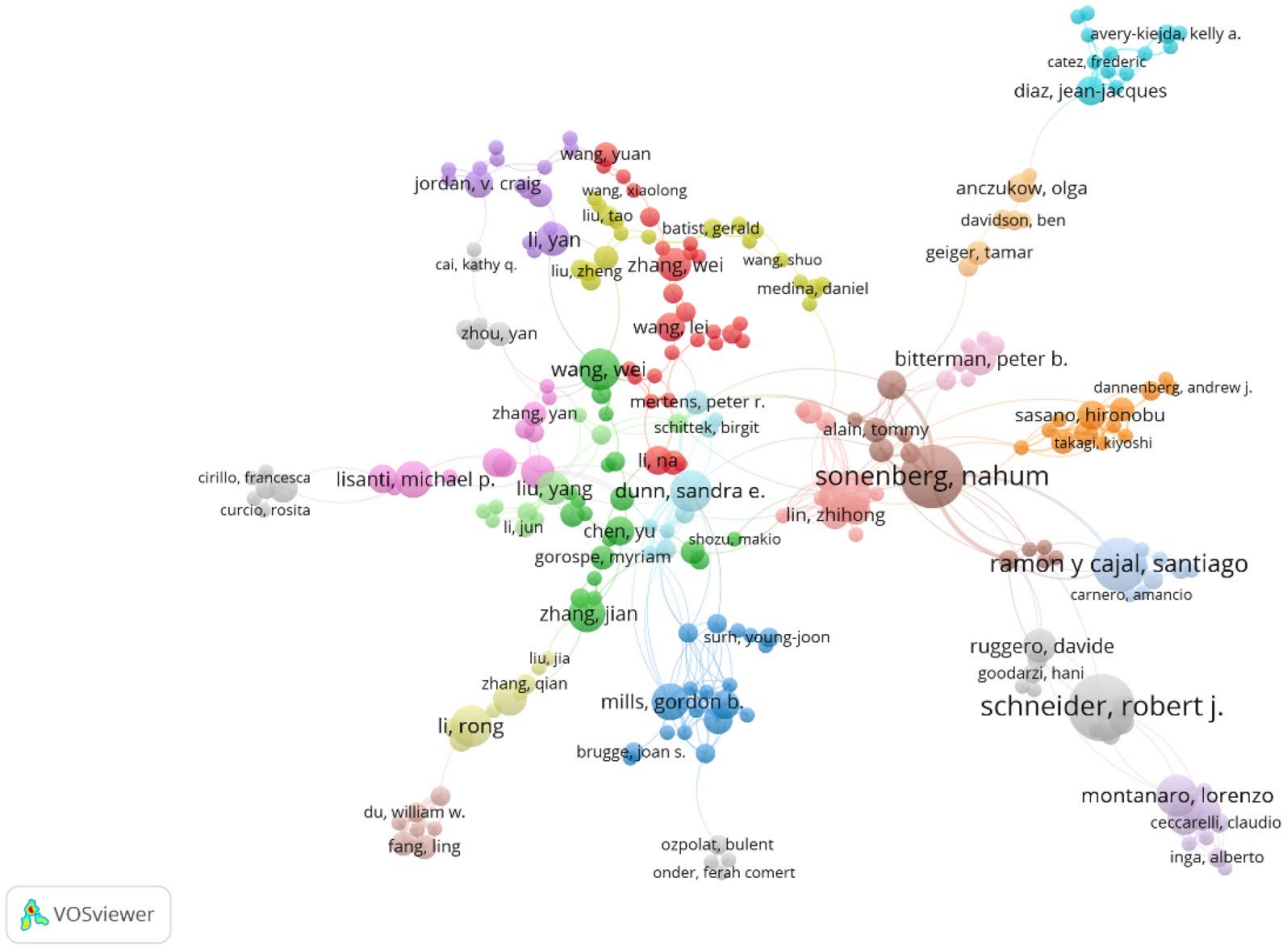
Collaboration network map of authors of breast cancer-related protein synthesis for the period 2003 to 2022

Fig. 6 reveals an intriguing hierarchy among the top authors in the field of breast cancer-related protein synthesis. The magnitude of their contributions varies considerably, with the preeminent author having a prodigious 15 publications and the tenth ranked author merely possessing a measly 8 publications. The commanding authority of Schneider, Robert J in this field is unquestionable, as evidenced by his top-ranking position with an impressive 15 publications to his name. The second and third positions are occupied by Sonenberg Nahum and Ramon y Cajal Santiago with 14 and 12 publications, respectively. The remaining authors are relatively less prominent, having published between 11 and 8 papers in this area of research.

However, a more comprehensive evaluation of the impact and significance of these publications would offer deeper insights into the exact nature and degree of each author’s contributions to the study of breast cancer-related protein synthesis. Indeed, each author may possess a unique expertise or research focus, contributing to the multifaceted nature of this field. Further investigation and analysis are necessary to fully comprehend the role and significance of these authors in advancing our understanding of breast cancer-related protein synthesis.

With respect to the authors’ contributions to breast cancer-related protein synthesis, it is noteworthy that Schneider, Robert J has made significant inroads in comprehending the complex landscape of transcriptional regulation and gene expression in breast cancer cells [111–115]. The top authors in the study of breast cancer-related protein synthesis are consummate experts in their field, with their notable contributions having furthered our understanding of the intricate molecular mechanisms that underlie the progression and metastasis of breast cancer. Their pivotal work has enabled the identification of new, potentially transformative targets for therapy and has played an instrumental role in advancing the development of innovative treatments for this pervasive and debilitating disease.

In our analysis of co-citation relationships between authors in the field of breast cancer-related protein synthesis, we found that the top eight most frequently co-cited authors were from a diverse range of countries and institutions. Among these authors, Gingras AC had the most co-citations (163), followed closely by Hanahan D with 153 co-citations and Bulun SE with 132 co-citations. Zhang Y, Graff JR, Ruggero D, Bartel DP, Menendez JA, and Sonenberg N were also highly co-cited, with 124, 123, 119, 118, 117, and 114 co-citations, respectively.

**Fig. 7:**
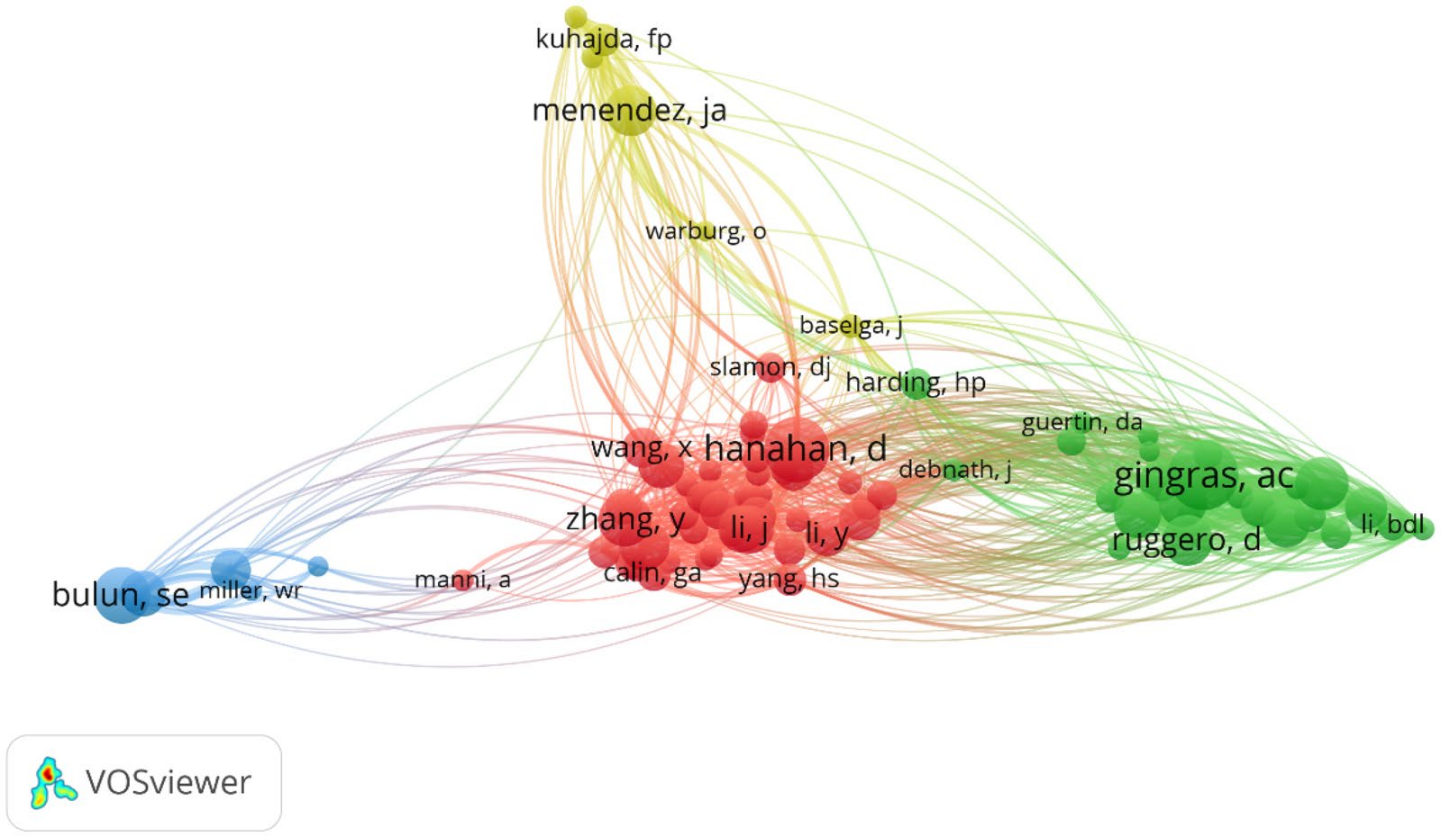
Co-citation network map of authors of breast cancer-related protein synthesis for the period 2003 to 2022

To better understand the co-citation relationships between these highly cited authors, we created a visualization (Fig. 7) that meticulously illustrates the intricate connections among authors who have garnered more than 50 co-citations. This figure highlights the most influential authors in the field and provides insights into the collaborative networks that have emerged among them.

### Keywords analysis

**Fig. 8:**
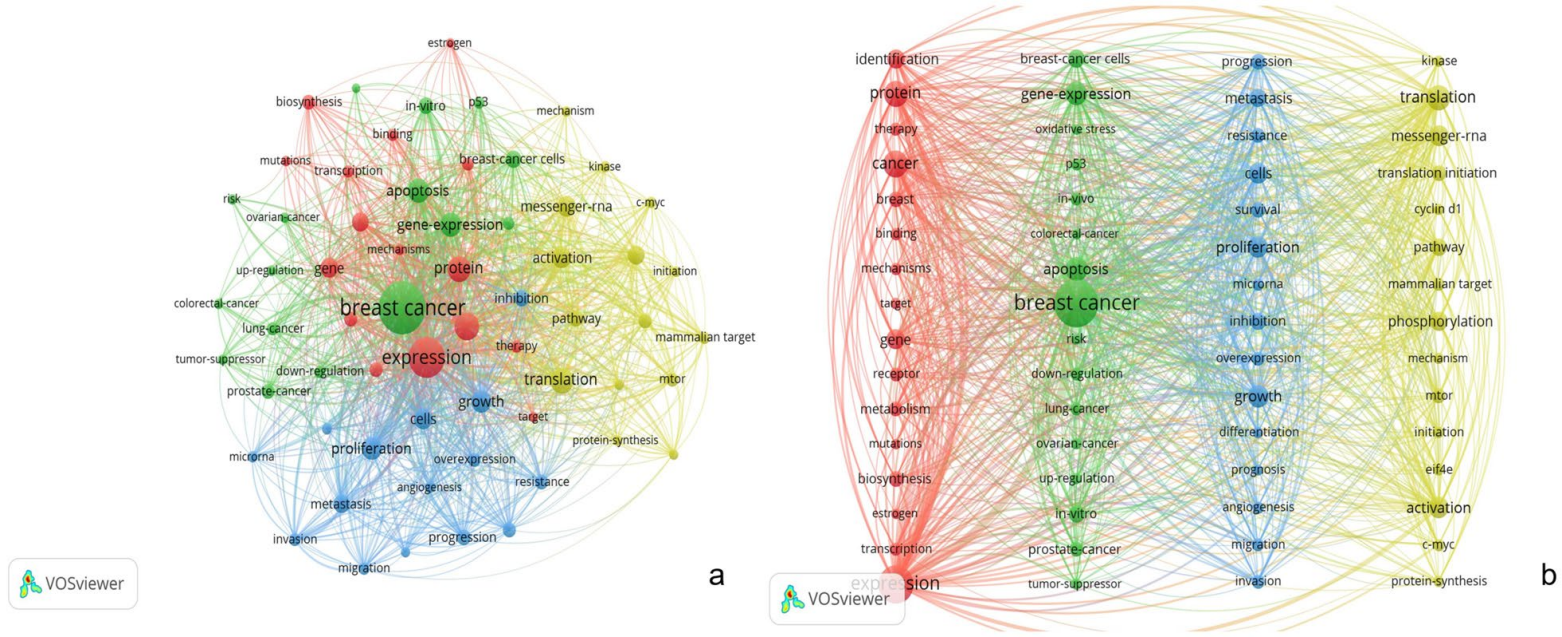
**a** Keywords network map of breast cancer-related protein synthesis for the period 2003 to 2022. **b** Keywords network map of breast cancer-related protein synthesis generated by VOSviewer were consolidated and segmented using Pajek software to divide topics with the same clusters in the same area

Figure 8a shows a network map of keyword co-occurrence for breast cancer-related protein synthesis. Following its generation, the obtained map underwent meticulous scrutiny and division utilizing the Pajek software. Employing a segmentation process, topics exhibiting akin clusters were deftly allocated to cohesive areas, thereby engendering a heightened sense of organization and a more comprehensive grasp of the underlying data (Fig. 8a). In this analysis, a keyword co-occurrence analysis was conducted to identify the most frequently appearing terms. The analysis included five keywords: “breast cancer” with 1339 occurrences, “expression” with 831 occurrences, “cancer” with 407 occurrences, “protein” with 358 occurrences, and “translation” with 350 occurrences. These results suggest that the analysis primarily focused on the relationship between breast cancer and protein synthesis, including gene expression, translation, and apoptosis. The aim of this analysis was to identify the most frequent keywords related to breast cancer-related protein synthesis and to gain insights into the relationship between breast cancer and protein synthesis.

The keywords cluster landscape (Fig. 8b) underwent synthetic knowledge synthesis, leading to the identification of 4 distinct themes and 17 Prevailing sub-categories, which are presented in Table 4.

**Table 4:** Results of the synthetic knowledge synthesis

Theme	Color	More frequent codes	Prevailing sub-categories
Synthesis and regulatory mechanisms of breast cancer-related proteins	Yellow	Translation (350), activation (254), messenger RNA (225), phosphorylation (198), pathway (151), translation initiation (123), mTOR (107), mammalian target (85), c-myc (75), kinase (68), protein synthesis (67), eIF 4 E (66), initiation (66), cyclin d 1 (65), mechanism (63)	Regulation of Protein Synthesis, mRNA Regulation and Translation Activation, Impact of Protein Synthesis in Breast Cancer Progression, Molecular Mechanisms and Signaling Pathways
Protein Synthesis and Cellular Processes in Breast Cancer Progression and Metastasis	Blue	Growth (274), proliferation (259), cells (232), metastasis (174), inhibition (155), progression (133), survival (125), resistance (118), overexpression (105), invasion (98), migration (82), angiogenesis (75), microRNA (71), differentiation (70), prognosis (65)	Cellular Processes in Breast Cancer Progression, Protein Synthesis and Cancer Cell Survival, Protein Synthesis and Differentiation, Prognostic Factors in Breast Cancer, Inhibition of Protein Synthesis in Breast Cancer
Apoptotic Regulation and Related Cancer Research in Breast Cancer Cells	Green	Breast cancer (1339), apoptosis (314), gene expression (302), breast cancer cells (169), in vitro (154), prostate cancer (114), in vivo (92), lung cancer (90), down-regulation (88), p 53 (81), up-regulation (66), ovarian cancer (65), risk (65), tumor suppressor (63), colorectal-cancer (59), oxidative stress (58)	Mechanisms of Apoptosis in Breast Cancer Cells, p53 Tumor Suppressor Function in Breast Cancer, Comparative Apoptosis Studies Across Different Cancer Types, Oxidative Stress and Breast Cancer Risk
Association of protein expression with breast cancer development	Red	Expression (831), cancer (407), protein (358), biosynthesis (124), breast (123), gene (205), identification (192), metabolism (120), binding (80), receptor (100), therapy (87), transcription (83), mechanisms (77), target (63), mutations (59), estrogen (58)	Protein Expression and Biosynthesis in Breast Cancer, Mechanisms of Protein Regulation in Breast Cancer, Protein Metabolism and Binding in Breast Cancer, Therapeutic Targeting of Protein Expression in Breast Cancer

#### Synthesis and regulatory mechanisms of breast cancer-related proteins (yellow)

Breast cancer-related proteins undergo complex processes of synthesis and regulation, influencing various aspects of cancer development and progression. Understanding the intricate molecular mechanisms and signaling pathways governing protein synthesis in breast cancer cells is crucial for identifying therapeutic targets and improving patient outcomes. Delving deeper into the regulation of protein synthesis may lead to potential biomarkers and more effective treatments for this devastating disease.

The process of translating mRNA into functional proteins is tightly regulated in cells, and dysregulation of this process can contribute to breast cancer development [116, 117]. Multiple levels of regulation, including transcriptional control, post-transcriptional modifications, and translational regulation, influence protein synthesis [118–120]. RNA-binding proteins and non-coding RNAs, such as microRNAs, long non-coding RNAs, and circular RNAs, play essential roles in post-transcriptional regulation, either enhancing or suppressing mRNA translation and affecting key proteins involved in breast cancer pathogenesis [121, 122].

The synthesis of specific proteins significantly impacts breast cancer progression, as dysregulation of oncogenic proteins, tumor suppressors, and proteins involved in cell proliferation, apoptosis, and metastasis is frequently observed in breast cancer cells [123, 124]. A study reveals that eukaryotic initiation factor (eIF) is overexpressed in breast cancer [125]. Notably, cell cycle regulatory proteins including CD4/6 and RB1 have been found to have a significant impact on the regulation of breast cancer cell proliferation [126, 127]. Understanding the mechanisms governing the synthesis of these proteins is crucial for developing targeted therapies [128]. Aberrant expression of upstream regulators, such as mTOR (mammalian target of rapamycin) and eIF4E (eukaryotic translation initiation factor 4E), has been linked to the development of aggressive breast cancer phenotypes, further emphasizing the significance of investigating protein synthesis pathways in breast cancer [129, 130].

Multiple signaling pathways participate in the regulation of protein synthesis in breast cancer, with the well-known PI3K/AKT/mTOR pathway being a key regulator of translation initiation, promoting the synthesis of proteins essential for cell growth and survival [131]. Dysregulation of this pathway is commonly observed in breast cancer and provides potential therapeutic targets [132].

Given the critical role of protein synthesis in breast cancer progression, it has become an attractive target for therapeutic intervention. Ongoing efforts to develop small molecule inhibitors of translation initiation factors and RNA-binding proteins offer promising avenues for personalized and targeted breast cancer therapies.

#### Protein synthesis and cellular processes in breast cancer progression and metastasis (blue)

Breast cancer is a disease with intricate pathogenesis, including gene mutations, epigenetic modifications, and abnormal signaling pathways [133–136]. Oncogenes and signaling pathways, such as the mTOR pathway, play vital roles in regulating protein synthesis and promoting cancer cell survival [137]. Targeting these pathways offers potential therapeutic opportunities to specifically target cancer cells [138, 139]. Moreover, disrupted cellular differentiation contributes to cancer development. Dysregulation of protein synthesis can impair cellular differentiation in breast tissue, leading to the accumulation of undifferentiated or dedifferentiated cells. These alterations contribute to cancer development and aggressive behavior [140]. Certain proteins, including Ki67, can serve as prognostic biomarkers, offering valuable insights for predicting disease progression and guiding treatment decisions [141, 142]. Targeting protein synthesis has emerged as a promising therapeutic approach for breast cancer treatment [143]. Small molecule inhibitors of translation initiation factors and ribosomal proteins have shown potential in preclinical studies. Additionally, targeting specific signaling pathways involved in protein synthesis regulation holds promise for personalized treatment strategies [144]. Understanding these mechanisms is crucial for developing effective treatments and improving patient outcomes in breast cancer management.

#### Apoptotic regulation and related cancer research in breast cancer cells (green)

Apoptotic signaling pathways in breast cancer cells are broadly categorized into intrinsic and extrinsic pathways, regulated by pro-apoptotic and anti-apoptotic proteins [145, 146]. Dysregulation of these pathways can lead to apoptosis resistance, contributing to breast cancer development [147, 148]. Genetic and epigenetic alterations, including mutations in tumor suppressor genes and microRNAs, play critical roles in apoptosis dysregulation [149, 150]. Apoptosis resistance poses a significant obstacle in breast cancer treatment, leading to therapy failure and disease recurrence. Several mechanisms underlie apoptosis resistance in breast cancer cells, such as the overexpression of anti-apoptotic Bcl-2 family members and the activation of survival pathways like PI3K/AKT and NF-κB [151–153]. Moreover, apoptosis resistance has been associated with an increased metastatic potential, further complicating therapeutic interventions. Therapeutic strategies targeting apoptotic pathways, such as BH3 mimetics and caspase activators, show promise in preclinical studies [154–156]. A comprehensive understanding of apoptotic dysregulation holds potential for personalized breast cancer treatment. Further research and clinical trials are needed to translate these findings into effective clinical practice.

#### Association of protein expression with breast cancer development (red)

Epigenetic dysregulation, encompassing DNA methylation and histone modifications, plays a pivotal role in driving significant changes in protein expression patterns within breast cancer cells [157–159]. These alterations in protein expression are further influenced by dysregulated protein metabolism, involving protein degradation and turnover, specifically occurring in cancer cells [160, 161]. Additionally, the metabolic reprogramming observed in cancer cells, necessitated by the high proliferation rates, exerts a substantial influence on protein expression levels [162, 163]. This intricate interplay between epigenetic dysregulation and altered protein metabolism not only contributes to the pathogenesis of breast cancer but also opens up potential targets for novel cancer therapies [164, 165].

**Fig. 9:**
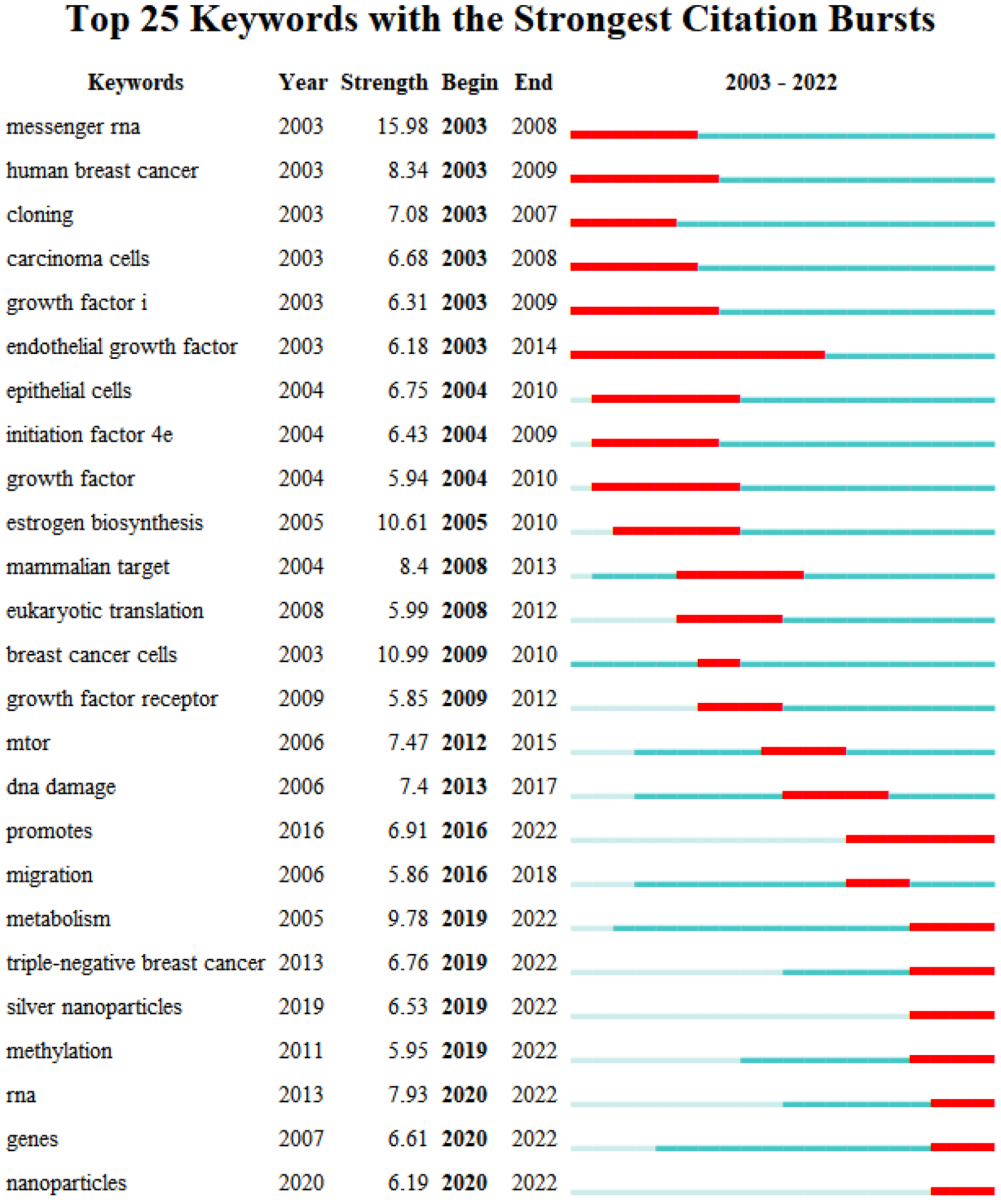
Keywords bursts of breast cancer-related protein synthesis for the period 2003 to 2022

Figure 9 shows the top 25 burst keywords, along with their start and end years and burst strengths. The keyword burst analysis of breast cancer-related protein synthesis reveals several keywords that have gained significant attention over the years. The burst keywords suggest that breast cancer-related protein synthesis research has focused on several areas, including messenger RNA, breast cancer cells, growth factors, and metabolism. Furthermore, the burst of keywords related to DNA damage and triple-negative breast cancer suggests that researchers have been investigating the mechanisms underlying breast cancer progression and developing new treatments. Additionally, the burst of keywords related to silver nanoparticles and methylation suggests that researchers have been exploring new approaches to cancer diagnosis and therapy.

**Fig. 10:**
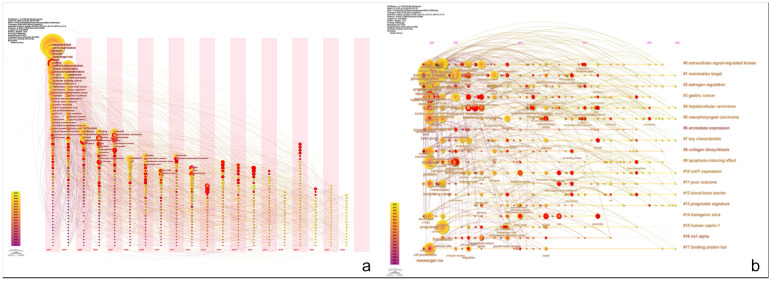
Keywords time zone (**a**) and time line (**b**) of breast cancer-related protein synthesis for the period 2003 to 2022

To further illustrate the historical process of breast cancer-related protein synthesis research, co-occurring keywords analysis was sorted by time zone and time line. This resulted in Fig. 10 which shows the evolution of research hotspots over time and provides insight into the trajectory of breast cancer-related protein synthesis research.

## Discussion

### General information of main findings

In this study, we have conducted a bibliometric analysis of the literature on breast cancer and protein synthesis to gain insights into the current research trends and focus areas in this field. From the provided research, several new knowledge insights were induced through the bibliometric analysis of breast cancer-related protein synthesis literature. Here are some key findings and new knowledge that emerged. Our findings reveal a steady increase in the number of publications in this area, with a significant rise observed after 2003. This indicates a growing interest and recognition of the importance of protein synthesis in breast cancer research.

The majority of the articles were published in oncology or biology-related journals, with the most publications in Journal of Biological Chemistry, Cancer Research, Proceedings of the National Academy of Sciences of the United States of America, and Oncogene.

The Journal of Biological Chemistry was found to be the most influential journal in the field. This journal has been a leading platform for research on protein synthesis in breast cancer, publishing groundbreaking studies on the molecular mechanisms underlying cancer cell growth, metastasis, and treatment resistance [166–170]. Other highly cited journals in the field include Cancer Research, Proceedings of the National Academy of Sciences of the United States of America, and Oncogene, which have played key roles in advancing our understanding of breast cancer biology and developing novel therapeutic approaches [171–177]. Overall, the analysis of journal influence provides insights into the scholarly landscape of breast cancer-related protein synthesis, highlighting the important role of top-tier journals in shaping the research agenda and driving scientific progress in this field.

The dual-map overlay provides valuable insights into the citation patterns and relationships between different fields of research, and can be used to identify key areas of overlap and potential research collaborations. Articles published in molecular biology genetics journals were primarily cited by articles in molecular biology immunology journals. This finding highlights the strong citation relationship between these two fields and suggests that research in molecular biology genetics is highly relevant to the field of molecular biology immunology.

Our text discusses the top institutions and countries in breast cancer-related protein synthesis research from 2003 to 2022. The top 10 institutions include five from the USA, three from China, and two from Canada. McGill University and Harvard University are the leading institutions in this field, with impressive publication and citation counts. The top 10 countries in breast cancer-related protein synthesis research, with the USA and China leading the pack with a significant number of publications. The data indicate that international collaboration is crucial in advancing research in this field, as evident from the diverse composition of countries in the top 10. Overall, the text emphasizes the global effort in researching breast cancer-related protein synthesis and highlights the importance of international collaboration in advancing research and development in this critical area.

The bibliometric analysis of breast cancer-related protein synthesis presented in this study provides valuable insights into the contributions of individual authors and their collaborative networks in advancing our understanding of this complex field. The hierarchy among the top authors based on their publication record reveals that Schneider, Robert J is the most prominent author, with 15 publications to his name, followed closely by Sonenberg Nahum and Ramon y Cajal Santiago. However, this study also acknowledges the need for a more comprehensive evaluation of the impact and significance of these publications to fully comprehend the role and significance of each author in advancing our understanding of breast cancer-related protein synthesis. This is important as each author may possess a unique expertise or research focus, contributing to the multifaceted nature of this field. Furthermore, the analysis of co-citation relationships between authors provides insights into the most highly cited authors in this field. Gingras AC, Hanahan D, and Bulun SE are the top three most frequently co-cited authors, with the remaining authors also highly co-cited. The visualization of the collaborative networks among these highly cited authors highlights the most influential authors in the field and their collaborative relationships.

### Hotspots and frontiers

The keyword analysis revealed that breast cancer, expression, translation, apoptosis, and gene expression were the most commonly researched topics. This implies that research in this field is primarily focused on understanding the relationship between protein expression in breast cancer and the development and treatment of tumors [25, 178–181].

The burst keyword analysis revealed several keywords that have gained significant attention over the years in the field of breast cancer-related protein synthesis. Among these, “messenger RNA,” “estrogen biosynthesis,” “breast cancer cells,” “DNA damage,” “metabolism,” and “RNA” had the highest burst strengths, indicating that they represent areas of active research and interest [182–187]. Examining the start and end years of each burst provides insight into the time periods during which each keyword experienced a surge in usage. For example, “messenger RNA” had a burst strength of 15.9835 from 2003 to 2008, while “metabolism” had a burst strength of 9.7751 from 2019 to 2022. These time periods may correspond to periods of active research in these areas. Additionally, analyzing the burst keywords may suggest potential research themes or areas of focus.

“Metabolism”—the burst of this keyword from 2019 to 2022 suggests that researchers are interested in the relationship between metabolism and breast cancer-related protein synthesis. This includes exploring the metabolic pathways involved in protein synthesis and their impact on breast cancer progression [182, 188].

“Silver nanoparticles”—the burst of this keyword suggests that researchers are exploring the use of silver nanoparticles in breast cancer therapy by targeting protein synthesis pathways. This study found that the nanoparticles showed significant induction of apoptosis in human breast cancer cells, possibly through oxidative stress-mediated ROS generation and loss of MMP, and were more effective than the aqueous extract [189]. Furthermore, El-Deeb et al. describes the synthesis and evaluation of the anticancer properties of biogenic silver nanoparticles produced using active metabolites from algae, which were found to have dose-dependent cytotoxic effects against different cancer cell lines, induce cellular apoptosis, and decrease tumor growth in a breast cancer model [190].

“Methylation”—the burst of this keyword indicates that researchers are investigating the role of DNA methylation in breast cancer-related protein synthesis, which may provide insights into new therapeutic strategies for breast cancer treatment [191–193].

The burst of “Nanoparticles” further indicates that researchers may be investigating the potential of nanoparticles for RNA delivery in breast cancer therapy. Shirangi et al. describes a system consisting of superparamagnetic iron oxide nanoparticles (SPIONs) decorated with silk sericin (SS NPs) for the targeted delivery of ROR1 siRNA, which demonstrated significant cell death and knockdown of ROR1 expression in TNBC cells both in vitro and in vivo, indicating potential as a therapeutic strategy for treating TNBC [194]. In addition, *Rubus fairholmianus* root extract was used to synthesize zinc oxide nanoparticles (RFZnO NPs), which showed cytotoxicity and induced apoptosis in MCF-7 breast cancer cells through a mitochondria-mediated caspase-dependent pathway, suggesting its potential use for cancer treatment [195].

In summary, analyzing burst keywords in breast cancer-related protein synthesis research can provide insights into emerging trends and areas of research interest. These findings could inform future research directions and highlight potential opportunities for advancing our understanding of breast cancer biology and developing effective treatments.

## Conclusions

From the provided research, several new knowledge insights were induced through the bibliometric analysis of breast cancer-related protein synthesis literature. Here are some key findings and new knowledge that emerged:

1. Research Growth Trend: this study revealed a steady increase in the number of publications related to breast cancer and protein synthesis, with a significant surge observed after 2003. This indicates a growing interest and recognition of the importance of protein synthesis in breast cancer research.
2. Key Research Journals: the analysis identified the most influential journals in the field of breast cancer-related protein synthesis, such as the Journal of Biological Chemistry, Cancer Research, Proceedings of the National Academy of Sciences of the United States of America, and Oncogene. These journals have been instrumental in publishing groundbreaking studies on the molecular mechanisms underlying breast cancer development, growth, and treatment resistance.
3. Leading Institutions and Countries: the research highlighted the top institutions and countries actively contributing to breast cancer-related protein synthesis research. McGill University and Harvard University emerged as leading institutions in this field, with the USA and China leading the pack among countries. The presence of diverse countries in the top 10 underscores the importance of international collaboration in advancing research and development in this area.
4. Influential Authors and Collaborative Networks: the analysis identified influential authors and their collaborative networks in the field. Schneider, Robert J, Sonenberg Nahum, and Ramon y Cajal Santiago were among the top authors. The co-citation analysis revealed significant collaboration among these authors, indicating the importance of joint efforts in advancing research.
5. Emerging Research Themes: the keyword analysis identified the most commonly researched topics in breast cancer-related protein synthesis, including breast cancer, expression, translation, apoptosis, and gene expression. Burst keyword analysis highlighted emerging research areas such as messenger RNA, estrogen biosynthesis, breast cancer cells, DNA damage, metabolism, RNA, silver nanoparticles, and methylation.
6. Potential Therapeutic Approaches: the burst of keywords related to silver nanoparticles and methylation suggested researchers are exploring new approaches to cancer diagnosis and therapy. Silver nanoparticles may have potential applications in breast cancer therapy by targeting protein synthesis pathways, while research on DNA methylation could provide insights into new therapeutic strategies for breast cancer treatment.

Overall, this bibliometric analysis provided valuable insights into the current research trends, focus areas, and collaboration networks in the field of breast cancer-related protein synthesis. The identification of influential authors, institutions, and emerging research themes can inform future research directions and contribute to the advancement of breast cancer diagnosis and treatment.

### Limitations

The use of bibliometric analysis has become increasingly popular in scientific research, as it provides a quantitative and objective approach to evaluate the productivity and impact of scientific research. However, it is important to note that bibliometric analysis has some limitations, this study only included publications from the WoSCC database, which may have missed out on some relevant publications from other databases. This could affect the comprehensiveness of the study’s findings. This study relied on citation analysis to identify the most influential publications. However, some recent high-quality publications might have been less cited and their impact underestimated, leading to potential citation bias. In addition, our research focused only on breast cancer-related protein synthesis and may have missed out on other important aspects of breast cancer research. This could limit the generalizability of the study's findings.

## Data Availability

The datasets used in this study were obtained from Web of Science. Interested parties can access the data by creating an account on the Web of Science website and conducting a search using the search terms provided in the Methods section of this paper.
